# Heparan Sulphate Glycosaminoglycan Chains Contribute to the Tethering of Coronal Factors and Are Important for Extracellular Vesicle‐Mediated Fibroblast Activation

**DOI:** 10.1002/jex2.70146

**Published:** 2026-05-09

**Authors:** Sara Veiga, Alex P. Shephard, Kate Milward, Alex Cocks, Félix Royo, Juan M. Falcon‐Perez, Aled Clayton, Jason P. Webber

**Affiliations:** ^1^ Tissue Microenvironment Group, Division of Cancer & Genetics, School of Medicine Cardiff University Cardiff UK; ^2^ Exosomes Lab, CICbioGUNE‐BRTA Derio Spain; ^3^ Centro de Investigación Biomédica en Red de enfermedades hepáticas y digestivas (CIBERehd) Madrid Spain; ^4^ IKERBASQUE, Basque Foundation for Science Bilbao Spain; ^5^ Institute of Life Science, Swansea University Medical School Swansea University Swansea UK

## Abstract

Extracellular vesicles (EVs) play a critical role in intercellular communication, yet the contribution of the EV corona and associated surface structures, such as heparan sulphate glycosaminoglycan (HS GAG) chains, to EV function remains poorly understood. In this study, we highlight a hitherto unknown requirement of HS GAG chains for the simultaneous delivery of a myriad assortment of growth factors by EVs. We demonstrate an attenuated function following enzymatic removal of HS GAG chains from the surface of prostate cancer (PCa)‐derived EVs, using heparinase III (HepIII). Our results confirm that digestion of HS GAG chains is specific and does not compromise EV integrity regarding size or tetraspanin expression. Enzymatic removal of HS GAG chains did, however, substantially altered the vesicular protein profile, reducing the expression of factors such as midkine, CYR61 and TFPI implicating HS GAG chains as a mode of tethering these factors to the EV surface. Importantly, EV‐associated HS GAG chains are required for functional delivery of such factors, resulting in successful activation of cellular signalling pathways for SCF, IGF‐1, midkine and VEGF in recipient fibroblasts. Furthermore, HS GAG chain removal attenuated EV‐induced fibroblast production of pro‐angiogenic factors VEGF and angiogenin as well as inflammatory factors VCAM‐1 and IL‐1α alpha/IL‐1F1, underscoring the role of vesicular HS GAG chains in mediating functional outcomes. These findings highlight the importance of EV surface HS GAG chains in growth factor delivery and signalling, providing new insights into the EV corona and its relevance in pathological processes relating to modulation of the tissue microenvironment.

## 1 Introduction

Cellular communication between cells within the tumour microenvironment is a key feature of tumour growth and disease progression. Extracellular vesicles (EVs) are a key component of this complex interaction and the nature of their influence on many diseases is the subject of intense investigation. For several solid cancers, including prostate cancer (PCa) the myriad roles of EVs are well documented and diverse. These include regulation of stromal (Webber et al. 2010) and leukocytic (Clayton et al. 2007; Wolfers et al. 2001; Zhang et al. 2025) cell phenotypes, as well as the local vasculature (Janowska‐Wieczorek et al. 2005; Luo et al. 2023; Krishn et al. 2020). Furthermore, EVs have been shown as drivers of tumour metastasis capable of priming secondary sites such as lymph nodes, bone and other organs in readiness for cancer cell colonisation (Peinado et al. 2012; Dai et al. 2019). Given the molecular complexity of EVs, defining a mode of action for these varied effects is a continuous challenge for this research field, as EVs have the capacity to target and influence recipient cells through a number of mechanisms (Alex et al. 2017; Belényesi et al. 2025).

The discovery that growth factors can be associated with EVs was made some time ago. Classic examples include FGF, VEGF (Taverna et al. 2003; Proia et al. 2008; Schiera et al. 2007; Thompson et al. 2013), TGF β1 (Clayton et al. 2007; Mitchell et al. 2008) and EGFR (Al‐Nedawi et al. 2008) among others. These present the concept of ligand delivery to recipient cell receptor(s) through vesicle‐mediated interactions, resulting in signal‐inducing process, and biological responses. The signalling pathways for many ligands are likely already well defined, and there are usually reagents available, such as small‐molecule inhibitors or antagonistic antibodies, to demonstrate signalling competency and consequences of signalling attenuation.

Among the controversies here, however, are considerations of the ultimate purity of the vesicle isolates used in such studies. Soluble mediators like these might be present as contaminants unless robust procedures such as gradient‐based centrifugations or affinity‐based techniques have been employed (Lamparski et al. 2002; Sharma et al. 2018). Furthermore, defining the nature by which such factors are tethered to the vesicle surface, forming a functional EV corona, or indeed identifying if such factor(s) are encapsulated within the vesicle lumen, remain a challenge. Such considerations are important to differentiate what could be considered as genuine vesicle‐association, as opposed to a non‐vesicular contaminant present in the isolate, and potentially mislead our understanding of mechanisms of vesicle activity (Théry et al. 2018; Welsh et al. 2024).

We have previously highlighted a role of PCa‐cell derived EVs in activation of stromal fibroblasts to a disease‐associated phenotype, with pro‐angiogenic function, and capable of driving in vivo tumour growth. This function of EVs was found to be dependent upon the delivery of TGF‐β1, tethered to the EV surface, to recipient stromal‐fibroblasts. This triggered predictable SMAD‐dependent and independent intracellular signalling events, resulting in fibroblast differentiation to a myofibroblast‐like phenotype. EV‐mediated fibroblast differentiation was abrogated when targeting TGF‐β1 receptors (Alk5) or using a TGF β1‐specific antibody antagonist (Webber et al. 2010). Intriguingly, however, the nature of this form of myofibroblast, in terms of its pro‐tumoural behaviours in vivo, could not be reproduced by using soluble TGF β1 as a fibroblast stimulus (Webber et al. 2015). Hence, vesicle delivery of TGF β1 is biologically distinctive, for reasons we do not yet fully understand. Similar effects may also be true for other vesicle‐associated factors.

We demonstrated that a proportion of vesicle TGF β is bound to a transmembrane heparan sulphate proteoglycan (betaglycan) (Webber et al. 2010, 2015). This and other heparan sulphate proteoglycans (HSPGs) comprise a core protein, decorated with heparan sulphate (HS) and chondroitin sulphate (CS) glycosaminoglycan (GAG) chains. Such HSPGs provide a low affinity, yet high‐capacity, binding of soluble factors which has been well documented (Piszczatowski et al. 2024). When exploring the roles of HSPG present on the plasma membrane of cells, there are several examples of soluble factors that become immobilised and bound to these molecules (Bishop et al. 2007; Sarrazin et al. 2011; Hayashida et al. 2022). Alternatively, HSPGs such as agrin (Kim et al. 2003) and perlecan (Chuang et al. 2010) can be located within the extracellular matrix where they bind FGF2 and FGF18, potentially stabilising ligand‐receptor interactions. HSPG‐association is also important for the physical handover of the soluble factor to signalling receptors, ensuring conformational advantage and enhanced signalling efficiencies (Filla et al. 1998; Yayon et al. 1991). Furthermore the roles of HSPGs such as syndecan 1 and syndecan 4 have been explored in the context of EV‐biogenesis and cargo loading (Baietti et al. 2012; Ghossoub et al. 2020) and HS GAGs implicated in uptake of EVs by recipient cells (Christianson et al. 2013; Song et al. 2023). The role of GAG‐chains associated with vesicles, and their functional relevance in terms of growth factor deliver, remains an interesting topic that has yet to be clarified. This is the focus of the current study.

Here, we demonstrate the use of enzymatic digestion as a tool to remove HS GAG chains and by doing so achieve a concomitant loss in HS GAG chain‐associated factors from the surface of small EVs. Such treatment does not impact EV integrity. We define a repertoire of classical soluble factors, which include chemokines and inflammatory factors, tethered to the surface of EVs by association to HS GAG chains. Furthermore, we demonstrate that the qualitative nature of EV‐triggered signalling within recipient fibroblasts is drastically altered upon HS GAG chain removal, resulting in an attenuated myofibroblastic response. Overall, the study extends our understanding of EV‐mediated growth factor tethering and delivery, emphasising the important and complex role of HS GAG chains in regulating EV corona constituents and function.

## 2 Results

### 2.1 Removal of EV‐Associated HS GAG Chains by Heparinase III Digestion Does Not Impact EV Structure or the Integrity of Transmembrane Proteins

**FIGURE 1:**
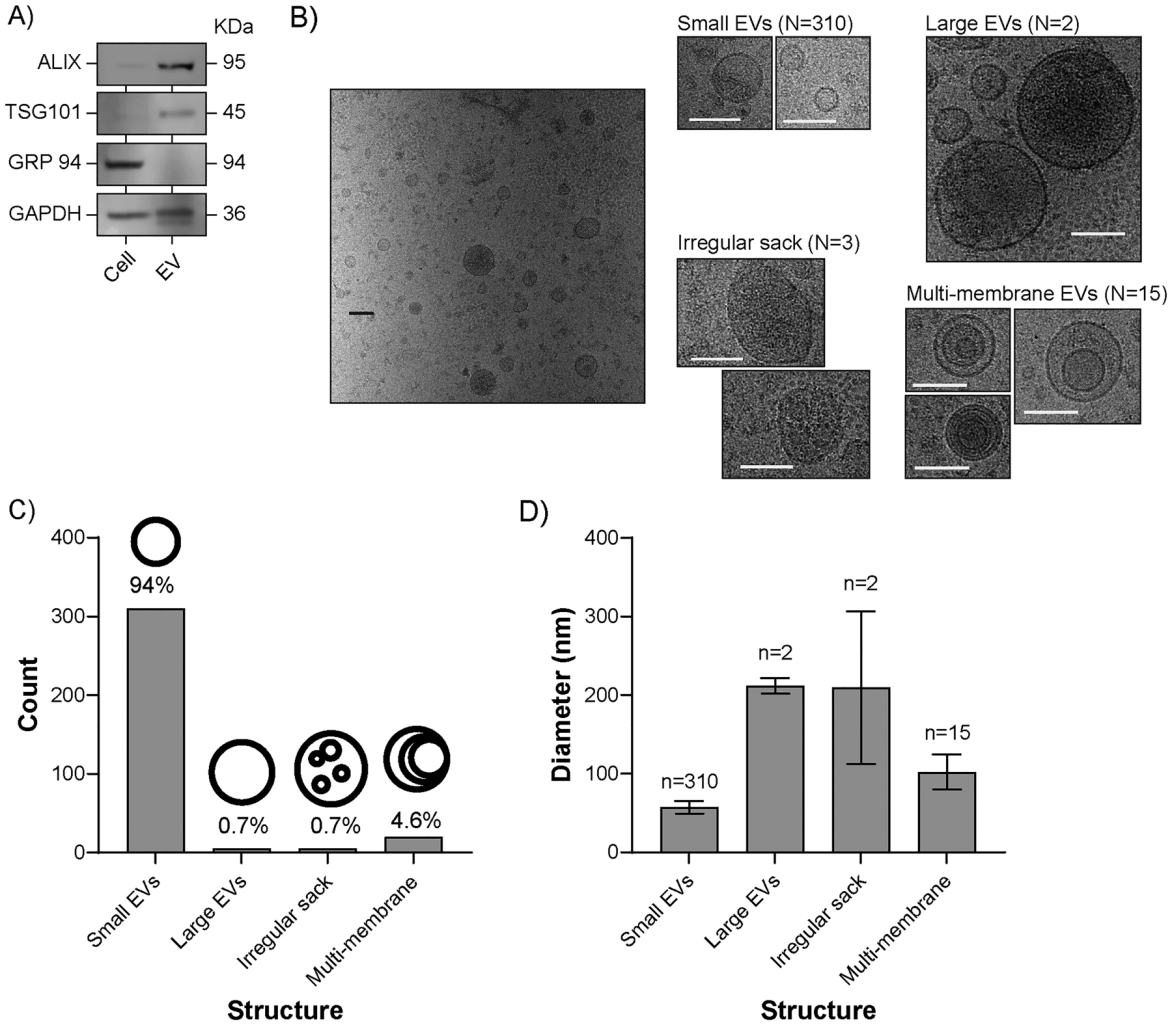
Characterisation of DU145 derived EVs. (A) DU145‐derived EV and cell lysates (20 µg/lane) were compared by SDS‐PAGE and Western blot using antibodies against ALIX, TSG101, GRP94 or GAPDH. (B) Isolated EVs were visualised by cryo‐electron microscopy (scale bar = 200 nm) and structures classified into arbitrarily assigned categories based on size and shape. The number of events analysed across all fields of view is shown. (C) Bars shown represent the total count of each EV structure, with a schematic to help identify the structure types and relative structure frequency shown as %. Bars represent the event counts. (D) Graph shows the mean ± SEM of the diameter (nm) for number of events measured. Data show a total of 329 structures counted across 48 fields of view.

EVs derived from PCa (DU145) cells were isolated through well‐established processes involving ultracentrifugation on a sucrose cushion (Théry et al. 2006). The EV product was assessed to examine the features of the isolate, in accordance with accepted guidelines (Théry et al. 2018; Welsh et al. 2024). As a proportion of total protein, EVs exhibited relative enrichment in endo‐lysosomal associated components such as ALIX and TSG101 compared to cell lysates (Figure 1A). In contrast, under these conditions, the endoplasmic reticulum‐associated protein GRP94 was undetectable on EVs, yet readily detected on an equivalent loading (by protein) of cell lysate. Cryo‐EM revealed a characteristic heterogeneous population of vesicular structures, where a defined lipid bilayer was evident. Some amorphous matter was also present within a limited number of fields, as well as some frost‐related artefacts (Figure 1B,C). Small unilamellar spherical structures of <100 nm comprised the majority (94%) of events present and are consistent with expectations of having purified small EVs. Other vesicle categories were arbitrarily assigned, purely on their morphology, based on a similar approach by Zabeo et al. (2017). Large unilamellar vesicles (>200 nm) and irregular‐shaped structures were rare (<1%), while occasional multilamellar structures were also evident, representing about 4% of the total events (Figure 1D). Together these data reflect the product from sucrose‐cushion isolations consisting of predominantly small EVs but also including other, rare, vesicular entities.

**FIGURE 2:**
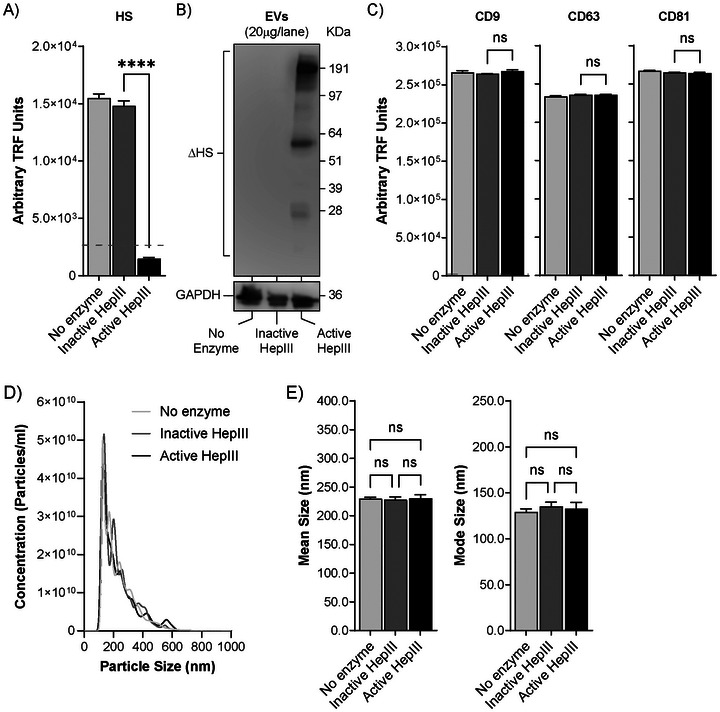
Confirmation of HepIII digestion. (A) EVs (1 µg/well), treated with active HepIII, heat‐inactivated HepIII or no enzyme, were immobilised onto protein‐binding plates and labelled either anti‐HS antibody or an IgM isotype control. Graph shows mean ± SEM of three independent experiments. ****p < 0.0001 control EVs versus active HepIII condition. (B) Vesicle samples (20 µg per lane) were subject to SDS‐PAGE and Western blotting, then subsequently detected using ΔHS and GAPDH specific antibodies. (C) EV tetraspanin expression was assessed by immobilisation of EVs (1 µg/well) onto protein‐binding plates and labelling with antibodies against either CD9, CD63 or CD81. Graph shows mean ± SEM of three independent experiments. ns = not significant. (D) EV size was assessed by NTA using the NanoSight NS300 platform with an overlay of individual size distribution histograms showing consistency across all conditions. (E) Bar graphs show mean ± SEM of mean and mode particle sizes, as assessed by NTA. ns = not significant.

DU145 EVs were treated with heparinase III (HepIII) to remove the HS GAG chains present on the EV surface, as described previously (Christianson et al. 2013; Webber et al. 2015). HepIII catalyses the eliminative scission of glycosidic linkages between N‐sulfated or N‐acetylated glucosamine (GlcNSO3or GlcNAc) and glucuronic acid (GlcA), resulting in the degradation of heparan sulphate, with minimal activity towards heparin. After washing by ultracentrifugation, successful HS GAG digestion was confirmed with antibody‐based methods. EVs were immobilised to protein‐binding ELISA‐plates and N‐sulphated glucosamine residues present on HS were detected using a time resolved fluorimetric method (anti‐HS; clone F58‐10E4). The treatment condition revealed a signal 10 times lower than the non‐digested controls, where HS was readably detected (Figure 2A). Non‐digested controls included the absence of enzyme and also a heat‐inactivated HepIII enzyme, which gave comparably high signals, indicating that the presence of enzyme in the system does not interfere with the capacity of detecting HS in this assay. Furthermore, western blotting was performed using an ΔHS antibody (clone F69‐3G10), which recognises the neo‐epitopes on proteoglycans left exposed by HepIII digestion. Consistent with our previous studies (Webber et al. 2015), the data reveal distinct bands of different molecular weights, corresponding to HSPG core proteins present on the EV surface, confirming successful digestion of HS GAG chains by HepIII (Figure 2B). Potential off‐target effects of HepIII digestion on the expression of classical EV‐related tetraspanins (CD9, CD63 and CD81) and vesicle size, indicative of general EV damage were assessed. HepIII digestion did not alter tetraspanin expression, detected by immuno‐assay (Figure 2C), neither did it impact vesicle size (Figure 2D,E). Overall, the data show that HepIII enzymatic digestion successfully removes HS from the EV surface without gross impact to EV surface proteins or vesicle size.

### 2.2 Identification of Proteins Tethered to the EV Surface by HS GAG Chains

**FIGURE 3:**
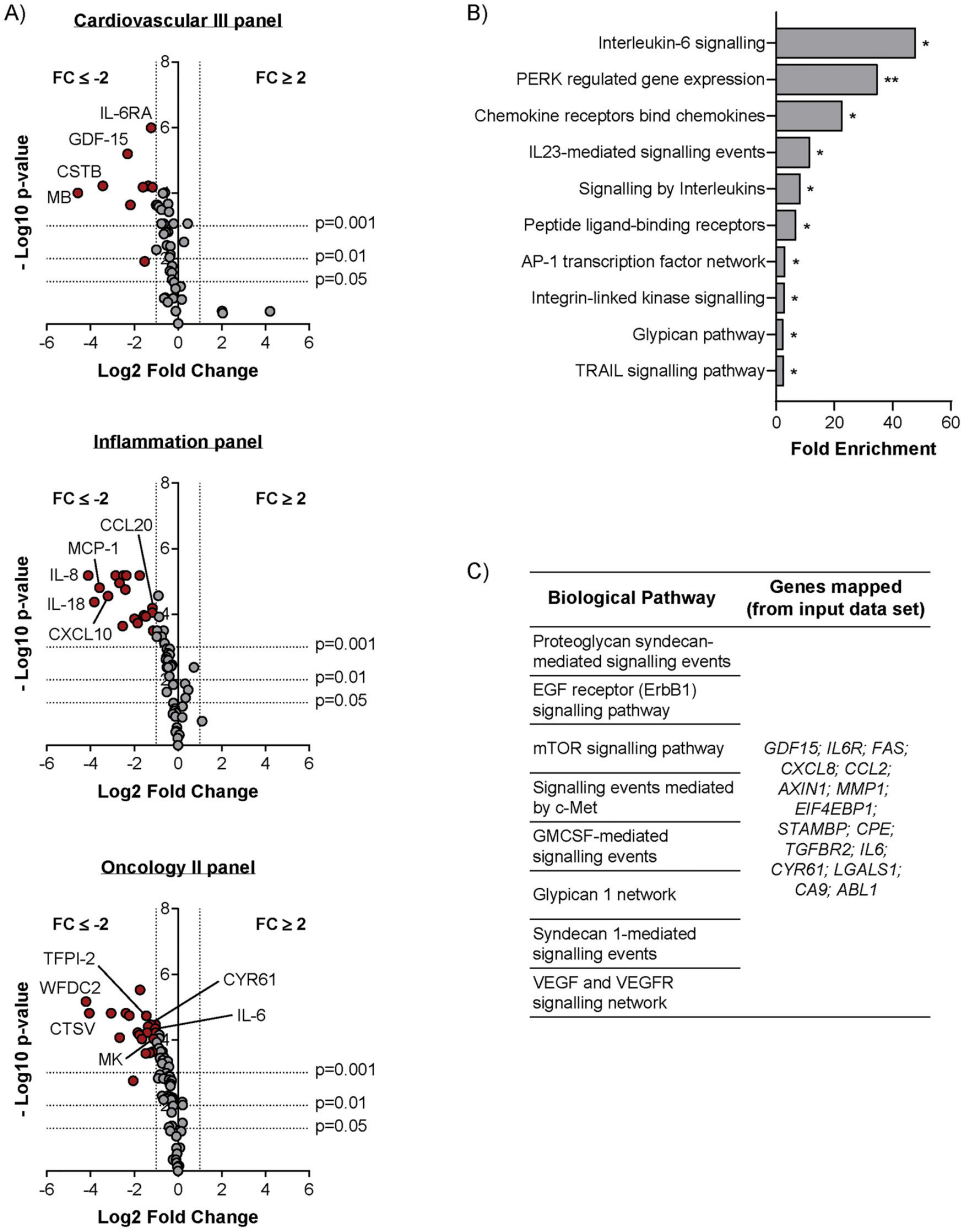
Enzymatic removal of HS GAG chains alters the protein profile of DU145 EVs. (A) A proximity extension assay (Olink) was used to compare proteins detected within lysates obtained from DU145 EVs treated with either active HepIII or with heat‐inactivated HepIII (control EVs). Volcano plots for the *Cardiovascular III*, *Inflammation* and *Oncology II* panels, show protein Log2 Fold Change plotted against –Log10 *p* value. Dotted lines indicate *p* values (*p* = 0.05, *p* = 0.01 and *p* = 0.001) and Fold Change (FC; FC = –2 and FC = 2). Statistical analysis was performed by *t*‐test, corrected for multiple testing using Benjamini–Hochberg adjustment. The 48 proteins which show a statistically significant (*p* > 0.05) decrease (FC ≤ 2) following digestion of HS are highlighted by red dots. Grey dots symbolise proteins detected, but outside this threshold criterion. Data shown represents the mean of *n* = 3 replicates. (B) Genes encoding for the 48 proteins differentially expressed between HepIII treated and control EV samples were analysed by FunRich software (version 3.1.4), highlighting the top 10 EV‐regulated biological pathways potentially impacted by HS GAG chain removal, ranked by fold enrichment. (C) List of gene names of EV proteins associated with biological pathways relating to proteoglycans, angiogenesis, cell proliferation and differentiation, identified by functional enrichment analysis and decreased following treatment with HepIII.

The protein profiles of EVs treated with either an active or a heat‐inactivated (control) form of HepIII were evaluated using highly sensitive, multiplex, Proximity Extension Assay (PEA) technology (Olink) to determine the impact of HS digestion on vesicle phenotype. Three panels were selected for this readout (*Cardiovascular III*, *Inflammation* and *Oncology II*), encompassing a total of 276 analytes implicated in the regulation of a variety of tissue responses. From the 276 analytes, 88 were excluded from our analysis as they were below the limit of detection on both HS‐digested and control EVs. Of the 188 analytes detected, four were duplicated across multiple panels, leaving 184 unique analytes to be investigated. We thus compared the analyte profiles of HS‐digested to control EVs. Volcano plots display the differences in analytes detected between the two conditions, revealing a total of 48 analytes with differential expression (*p* < 0.05 and a fold‐change equal or greater to 2) (Figure 3A). In all cases this was a reduction in growth factor expression levels, with no examples of heightened expression. HepIII‐mediated loss of HS GAG chains is therefore accompanied by a concomitant reduction in the detection of these 48 analytes EV‐associated, with the greatest number of impacted proteins being present within the *Oncology II* panel (22 proteins), followed by the *Inflammation* panel (18 proteins) and finally the *Cardiovascular III* panel (9 proteins) (Table S1). Since the detection of only 48 proteins was altered following digestion with HepIII, leaving 136 proteins unaffected, this suggests that  enzymatic treatment is restricted to a subset of proteins and does not comprehensively remove all detectable proteins, and hence there is some specificity in terms of growth factor association to HS GAG chains.

The gene names of the 48 proteins identified were input into FunRich software (version 3.1.4) (Pathan et al. 2015) to perform functional enrichment analysis to explore the potential EV‐regulated biological pathways which may be impacted by HS GAG chain removal. The number of biological pathways identified was extensive; the 10 pathways showing greatest fold enrichment are shown (Figure 3B), with further information on the top 50 most enriched biological pathways, and associated genes, also provided (Table S2). Interleukin and chemokine signalling pathways were amongst the most enriched, as well as several receptor related processes, which indicate that vesicular HS may play a key role in delivery of factors involved in cellular responses. Importantly, we identified other pathway associations with elements related to proteoglycans, angiogenesis, cell proliferation and differentiation, suggesting that these pathways may be, at least partially, regulated by HS GAG chains present on the surface of EVs (Figure 3C). Interestingly, such pathways (e.g., *Proteoglycan Syndecan‐mediated signalling events*, *EGF receptor (ErbB1) signalling pathway*, *mTOR signalling pathway*, *VEGF and VEGFR signalling network*, etc.) all shared the same 16 proteins, highlighting the potential significance of these factors for disease‐promoting functions of cancer‐derived EVs.

**FIGURE 4:**
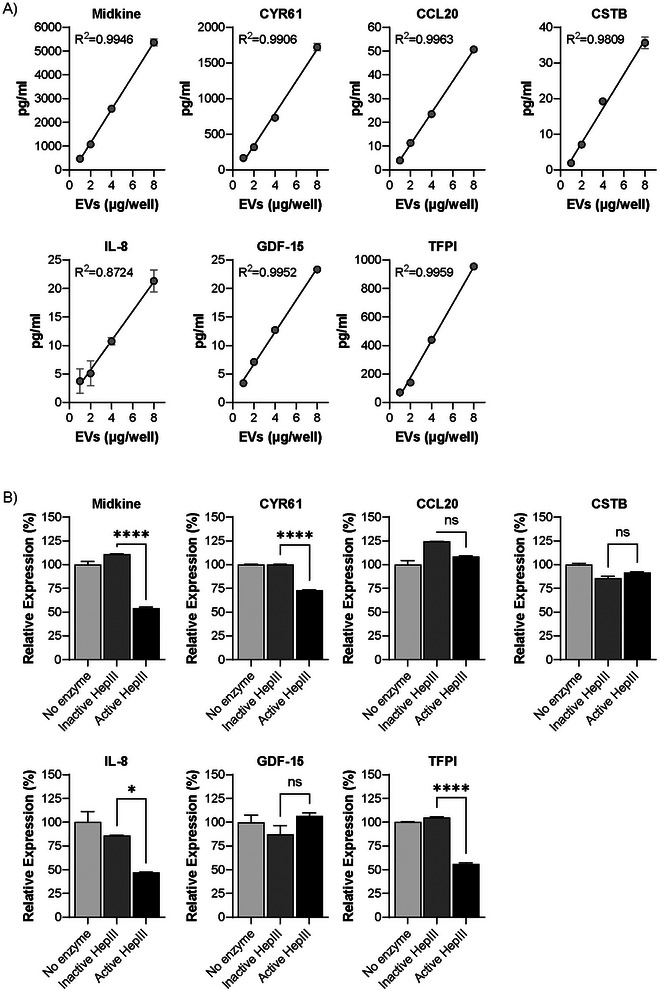
Removal of HS GAG chains results in altered detection of candidate proteins present on the EV surface. (A) Intact, non‐lysed, DU145‐derived EVs were assayed by ELISA. The graphs show mean ± SEM of best fit curve and *R* ^2^ values for candidate proteins assessed within an increased EV input, from a single EV isolate. (B) 4 µg of EVs treated with either active HepIII, heat‐inactivated HepIII or no enzyme were assayed by ELISA to quantify protein detection. The bar graphs show mean ± SEM of *n* = 3. ****p < 0.001; **p < 0.01; *p < 0.05; ns = not significant.

To confirm some of the array‐based identifications, and to quantify analyte abundance within the EV preparations before and after HS GAG removal, we used traditional ELISA‐like methods. A handful of candidate proteins were selected based on the magnitude of differential expression. The linear association between candidate protein signal detected and increasing EV dose, from a single isolation, was used to demonstrate assay performance and absence of interfering factors (Figure 4A). To strengthen the association of EV‐surface HS GAG chains with candidate proteins, EVs were not lysed prior to ELISA‐based protein detection. Furthermore, while EVs from different isolations, do not consistently produce the same detectable amount of protein, the trend between distinct isolations remains the same (Figure S1A).

The factors midkine, CYR61 and TFPI were most readably detectable at the EV surface with a mean of 85.7pg, 27.5pg and 11.7pg, respectively, detected per 1 µg of input EVs (Table 1). CXCL10, MCP‐1 and IL‐18 were also tested but signal detection was below limits of detection using traditional ELISA technologies (data not shown). As evidence that removal of HS GAG chains from the EV surface has an impact on the level of growth factors detectable, midkine, CYR61, IL‐8 and TFPI levels were significantly reduced following HepIII treatment, whereas the presence of heat‐inactivated HepIII had a weaker or negligible effect (Figure 4B). The detection of CSTB, CCL20 and GDF‐15 were also assessed in this regard, but the data forthcoming was inconsistent between repeat experiments; and hence we remain cautious about their HS GAG chain association on EVs (Figure S1B). Collectively, this data demonstrates that many candidate proteins identified by PEA are associated with the intact EV surface. Furthermore, these data confirm that midkine, CYR61, IL‐8 and TFPI are tethered to the EV surface by HS GAG chains.

**TABLE 1:** Concentration of candidate proteins detected per 1ug of input EVs.

Proteins detected	Midkine	CYR61	CCL20	CSTB	IL‐8	GDF‐15	TFPI
Concentration (pg/µg of EVs)	85.7	27.5	0.9	0.45	0.23	0.31	11.7

### 2.3 EV‐Associated HS GAG Chains Play a Key Role in EV‐Mediated Fibroblast Differentiation and Receptor Tyrosine Kinase Activation

**FIGURE 5:**
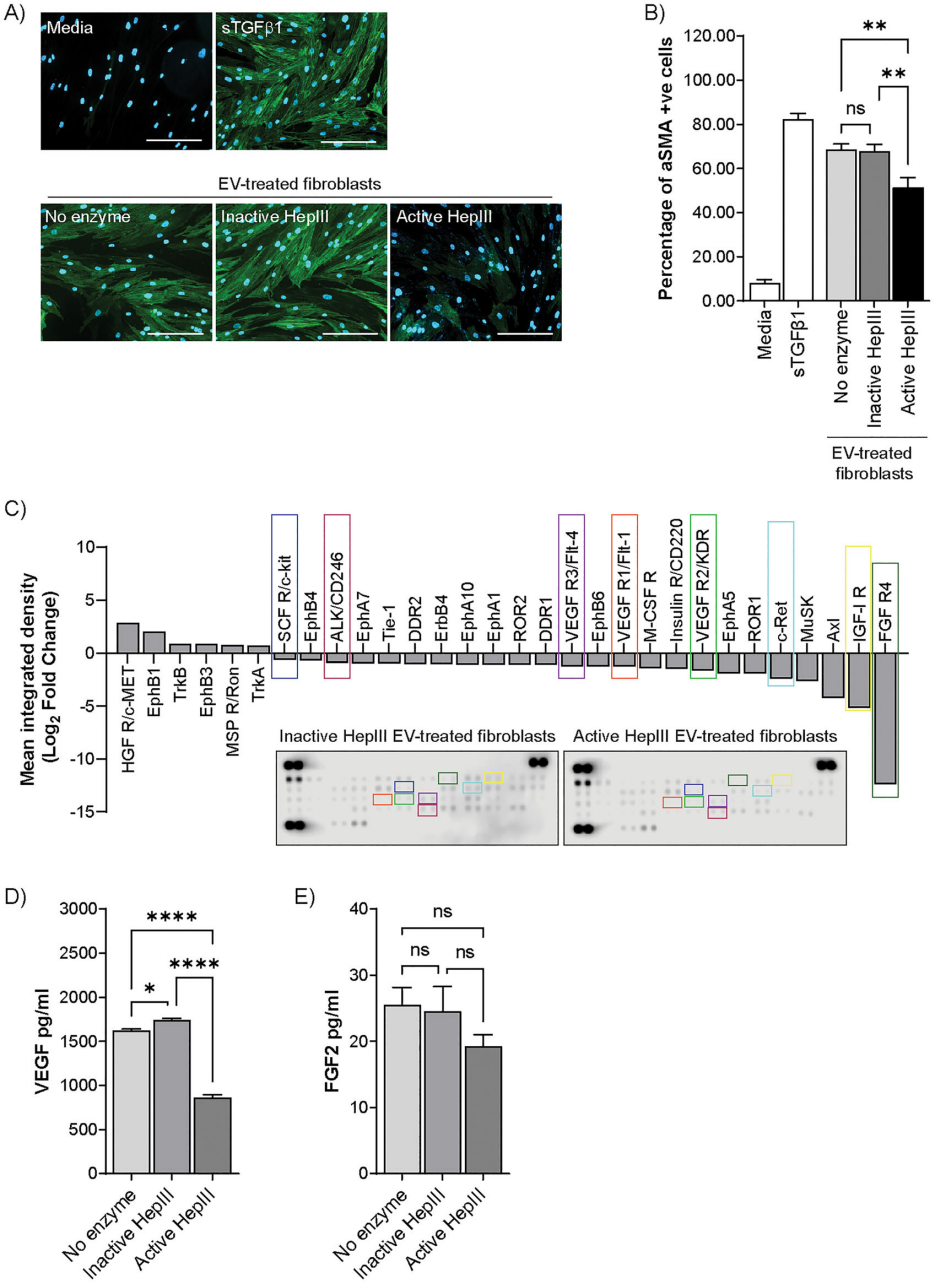
HS GAG chains are required for EV‐mediated fibroblast differentiation and receptor activation. (A) Fibroblasts were treated for 72 h with either media alone, sTGF‐β1 (1.5 ng/mL) or EVs (200 µg/mL). EVs were treated with heat‐inactivated HepIII, active HepIII or no enzyme. Cells were fixed, immuno‐labelled for αSMA (green) and nuclei stained with DAPI (blue), prior to visualisation by fluorescence microscopy. (B) Bar graph shows mean percentage ± SEM of αSMA‐positive cells, based on ≥9 fields of view from triplicate wells per condition. **p < 0.01, ns = not significant, one‐way ANOVA with Tukey's multiple comparison test. (C) A phospho‐RTK Proteome Profiler array was performed on cell lysates from fibroblasts incubated for 2 h with EVs treated with either active or heat‐inactivated HepIII enzyme. Densitometry‐based analysis shows receptors with at least 1.5‐fold change in level of phosphorylation following fibroblast incubation with active HepIII‐treated EVs compared with inactive HepIII‐treated EVs. Colour boxes reveal some HS GAG chain‐dependent differences in receptor phosphorylation, identifying analytes on the membranes and corresponding waterfall plot. (D, E) EVs treated with active HepIII, heat‐inactivated HepIII or no enzyme (4 µg per condition) were assayed by ELISA to quantify VEGF (D) and FGF‐2 expression. The bar graphs show mean ± SEM of *n* = 3. ****p < 0.001, **p < 0.01, *p < 0.05; ns = not significant.

The capacity of DU145‐derived EVs to induce fibroblast differentiation has been previously reported by our group (Chowdhury et al. 2015; Webber et al. 2010), with similar EV function observed in other settings (Borges et al. 2013; Mazumdar et al. 2020). Here we explored whether HS GAG chain‐deficient EVs retained the ability to induce the expression and organisation of alpha smooth muscle actin (αSMA) stress fibres in recipient fibroblasts, indicative of differentiation to a tumour‐supporting myofibroblast‐like phenotype (Webber et al. 2015). Fibroblasts stimulated with DU145‐derived EVs (not subject to HepIII digestion; No enzyme), or sTGF‐β1, are able to induce αSMA expression (Figure 5A). Fibroblasts incubated with HS‐deficient EVs (active HepIII), show a weaker induction of αSMA, visible by microscopy, with a notable decrease in the percentage of cells containing αSMA stress fibres (green) around the nuclei (blue) (Figure 5B). An additional control, consisting of fibroblasts incubated with DU145 EVs treated with heat‐inactivated HepIII (Inactive HepIII), resulted in a phenotype comparable to that induced by Control EVs (No enzyme) and sTGF‐β1. This further supports the suggestion that EV‐mediated fibroblast differentiation is at least partially regulated by HS GAG chains on the EV surface. Furthermore, we hypothesised that this lost EV function was the result of a reduced capacity for EV‐mediated growth factor delivery to recipient fibroblasts. To test this, fibroblasts were incubated with 200 µg/mL of HS‐deficient EVs or respective controls for 2 h, and phosphorylation of 49 human receptor tyrosine kinases was examined using a low‐density protein array.

The phosphorylation profile of EV‐stimulated fibroblasts triggered a marked activation of a repertoire of receptors. This profile of activated receptors was very similar to that obtained when using EVs treated with inactive HEPIII enzyme, indicating that the presence of HEPIII in the system did not contribute to any notable signalling events or enhancement of any EV effects (Figure S2). However, HS‐deficient EVs (Active HepIII treated) showed reduced capacity to trigger phosphorylation of several receptors compared to those activated by Control EVs (Inactive HepIII treated). Affected receptors included FGF R4 and IGF‐1 R, as well as receptors from the VEGFR family (Figure 5C). Consistent with these results, the expression of VEGF and FGF2, measured by ELISA, was lower in EVs lacking HS GAG chains (due to treatment with active HepIII) compared with Control EVs (Figure 5D). Differences in FGF2 expression did not reach statistical significance (Figure 5E). The activation of other receptors such as c‐kit, IGF‐1 R and ALK/CD246 were also altered following loss of HS GAG chains. Ligands for these receptors, SCF (c‐kit), IGF1 (IGF‐1 R) and midkine (ALK/CD246), where previously identified in the Olink analysis and shown to be reduced following digestion of HS GAG chains (Table S1) with reduced midkine expression confirmed by ELISA (Figure 4B). These experiments demonstrate that removal of HS GAG chains from the EV surface has a detrimental impact on functional delivery of certain growth factors to recipient fibroblasts, illustrated here by an attenuated ability to induce fibroblast activation and receptor phosphorylation.

EV‐mediated fibroblast activation likely requires simultaneous delivery of a multitude of factors. This would account for our previous demonstration EVs, but not sTGF‐β1 alone, were required for induction of a fibroblast phenotype capable of supporting prostate tumour growth in vivo (Webber et al. 2015). Such factors could be present on the EV surface or contained with the EV lumen, requiring EV uptake for delivery. We therefore explored the role of HS GAG chains, present on the EV surface, in EV uptake by recipient fibroblasts (Figure S3). EVs were fluorescently labelled with AlexaFluor‐conjugated C5 maleimide dye and any excess, unbound, dye was removed prior to adding EVs to fibroblast cultures for 1 h. Here, HS‐deficient EVs (active HepIII) digestion), showed no noticeable reduction in uptake by recipient fibroblasts, compared to control EVs not subject to HepIII digestion (No enzyme) or those EVs treated with heat‐inactivated HepIII (Inactive HepIII) when visualised by fluorescent microscopy (Figure S3A). Importantly, the absence of fluorescent puncta in the dye control suggests successful labelling of EVs with our dye, and removal of non‐EV contaminants prior to uptake experiments. EV‐uptake was also quantified by flow cytometry, further confirming that removal of HS GAG chains does not grossly affect EV‐uptake by recipient fibroblasts, as shown by the percentage of EV‐positive fibroblasts when comparing HS‐deficient EVs to control EVs with intact HS (Figure S3B). Furthermore, the difference in mean fluorescence intensity between conditions is small. The presence of the heat‐inactivated enzyme in the system, however, seemed to negatively impact EV uptake (Figure S3C). The reasons for this remain unclear and are likely related to the technical challenges of these experiments, in terms of absolute quantification and normalisation of the EV concentration added to the recipient cells. Importantly, this data suggests that HepIII‐treated EVs remain competent in gaining cell entry.

### 2.4 Cytokine Production Is Altered in Fibroblasts Incubated With HS GAG Chain‐Deficient EVs

**FIGURE 6:**
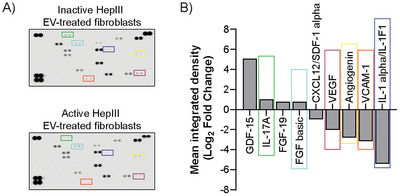
HepIII digestion of EV‐associated HS GAG chains results in an altered fibroblast cytokine production in response to PCa‐derived EVs. (A) A human cytokine Proteome Profiler array was performed on cell lysates from fibroblasts incubated for 72 h with EVs treated with either active or heat‐inactivated HepIII enzyme. Fibroblasts were incubated with Golgi‐Stop and Golgi‐Plug 18 h before lysis, to prevent cytokine secretion. (B) Densitometry‐based analysis shows cytokines with at least 1.5‐fold change in level of detection following fibroblast incubation with active HepIII‐treated EVs compared with inactive HepIII‐treated EVs. Colour boxes reveal some HS GAG chain‐dependent changes in cytokine production, identifying analytes on the membranes and corresponding waterfall plot.

To further explore the biological impact of enzymatic removal of HS GAG chains on EV‐mediated regulation of fibroblast function, cytokine production from recipient fibroblasts was evaluated following incubation with EVs, using a human cytokine array. Fibroblasts were incubated for 72 h with 200 µg/mL of either HS GAG chain‐deficient EVs (treated with active HepIII) or control EVs (treated with heat‐inactivated HepIII or no enzyme). Control conditions show that EVs which had not been subject to enzymatic digestion of HS GAG chains could induce fibroblast cytokine production (no enzyme EV‐treated fibroblasts) compared to untreated fibroblasts (Figure S4A,B). Importantly, there was a high degree of similarity in EV‐induced fibroblast cytokine production when comparing non‐digested EVs to EVs treated with a heat‐inactivated HepIII. Both conditions resulted in some increase in the de novo production of cytokines such as ICAM, uPA, VEGF, VCAM, FGF‐2 and angiogenin (Figure S4C). Enzymatic removal of HS GAG chains from EVs resulted in a lower production of angiogenesis‐promoting factors VEGF, VCAM1 and angiogenin in fibroblasts, and an apparent increase in inflammatory cytokines GDF‐15 and IL‐17A, when compared with control EVs (treated with heat‐inactivated HepIII) (Figure 6). Taken together the data show that digestion of the HS GAG chains from the EV surface, and consequent reduction in the growth factors they carry, results in a reduced capacity for PCa‐derived EVs to activate fibroblasts to a disease‐associated phenotype, with pro‐angiogenic function.

## 3 Discussion

It is becoming increasingly important to understand the constituents of the EV surface and the surrounding corona if we are to elucidate mechanisms by which EVs can interact with cells in the surrounding microenvironment (Buzás et al. 2018). Here, we show that removal of HS GAG chains from EV surface, by means of enzymatic digestion using HepIII, is specific and does not impact EV integrity with regard to tetraspanin expression or EV size. Modifications to EV HS GAG chains did, however, have a substantial impact on the molecular composition of EVs, highlighting the importance of HS GAG chains in the functional role of EVs within biological systems.

Our approach also revealed that HS GAG chains are required for the tethering of select growth factors to the EV surface. This included factors such as midkine, CYR61, IL‐8 and TFPI whose expression within PCa‐derived EVs was reduced following enzymatic digestion of EV‐associated HS GAG chains by digestion with HepIII. Despite the association of these factors with heparin/heparan sulphate being previously known (Sumi et al. 2002; Kadomatsu et al. 2013; Chen et al. 2000; Ho et al. 1997; Grzeszkiewicz et al. 2002; Ori et al. 2011), their association with HS GAG chains at the EV surface was hitherto unknown. We are yet to understand the levels of EV‐associated factors that are required for successful signalling events. Whilst HepIII digestion resulted in reduced expression of factors such as midkine and TFPI, by around 50%, the reduction in expression of CYR61 was much more modest at approximately 27%. Our previous studies into EV‐mediated delivery of TGF‐β1 (Webber et al. 2010) indicate that the mode of delivery is important with a 30% loss of TGF‐β1, tethered to the EV surface by the HSPG betaglycan, being sufficient to abrogate EV‐mediated fibroblast differentiation.

The mechanism by which factors such as midkine, CYR61, IL‐8 and TFPI become incorporated into EVs is not known. Due to the role of HSPGs such as syndecan 1 and syndecan 4 in EV biogenesis and cargo loading (Baietti et al. 2012; Ghossoub et al. 2020), it is possible that the incorporation of such factors could occur prior to vesicle secretion. Alternatively, factors could be incorporated into EVs while within the extracellular environment. In a past report, incubation of EVs with excess recombinant TGF‐β1 did not result in an elevation of TGF‐β1‐associated to EVs (Clayton et al. 2007), suggesting that loading most likely takes place during biogenesis rather than capturing factors present in the extracellular environment. In contrast, other studies have reported that bovine‐derived proteins, such as albumin, may adsorb onto EV surface and influence EV corona composition (Urzì et al. 2024; Liam‐Or et al. 2024). Culture of cells in the presence of bovine serum is, therefore, a limitation of the current study. Determining where and how EV loading happens will be challenging and might require the focus on a specific growth factor as opposed to a general and complex model such as this system. Our functional enrichment analysis, revealing that proteins with attenuated expression following removal of HS GAG chains by HepIII were often associated with proteoglycan‐mediated signalling pathways (e.g., *Glypican 1 pathway/network* and *Syndecan 1‐mediated signalling events*), is reassuring. Interestingly, associations with receptor signalling and inflammation were also identified. Regardless of the limitations of the analysis, these data support our endeavours to better understand the EV heparome and its functional relevance in pathological processes.

An important highlight of this study is the observation that HS GAG chains, on the surface of PCa‐derived EVs, are important for the functional delivery of growth factors, and subsequent phosphorylation of receptor tyrosine kinases, on recipient fibroblasts. Incubation of fibroblasts with HS GAG chain‐deficient EVs (by treatment with HepIII) resulted in attenuated phosphorylation of several receptor tyrosine kinases compared to those activated in response to control EVs (treated with heat‐inactivated HepIII). Amongst the receptors where phosphorylation was impacted following digestion of HS GAG chains were the SCF receptor (c‐kit), insulin‐like growth factor 1 receptor (IGF‐1R), midkine receptor (ALK/CD246) and VEGF receptor (VEGFR). Additionally, we identified several receptor tyrosine kinases that showed increased phosphorylation following fibroblast incubation with HS GAG chain‐deficient EVs. Such receptors include HGF R (c‐MET), EphB1 and TrkB, suggesting that factors which negatively regulate these receptors may be associated with vesicular HS GAG chains, and its removal upon digestion allows receptor activation to occur. Whilst an attenuation in EV‐mediated RTK‐activation was often accompanied by a reduced expression of the associated growth factor (e.g. VEGF) on the EV surface, we cannot rule out a possible role of EV‐associated HS GAGs activating receptors directly. HS has been previously implicated in modulation of immune responses, including toll‐like receptor signalling and cytokine production (Duni et al. 2021; Chandra et al. 2025). Investigation into the ability of EV‐associated HS GAGs to signal directly would, however, require careful consideration of the specificity of the method chosen. A requirement for EV uptake to ensure full EV activity should also not be overlooked. Our findings indicating that PCa‐derived EV uptake occurred independently of HS GAGs was surprising, especially considering past studies (Christianson et al. 2013; Song et al. 2023). It is, however, important to note that these studies, and several similar studies, attempt to abrogate HS function during the incubation of recipient cells with EVs. Such treatments will simultaneously target both EV and cell‐associated HS. In our study, we specifically target on EV‐associated HS GAGs, removing HepIII prior to incubation of recipient cells with EVs. Therefore, whilst our data suggest EV‐associated HS GAGs are not required for EV uptake by recipient cells, we cannot rule out a requirement for cell‐associated HS GAGs. Irrespective of uptake, we show that EV‐associated HS GAGs are required for regulation of fibroblast activation and subsequent function. Collectively, these findings establish a relationship between intercellular signalling events and the EV corona, whilst shedding light on potential functional relevance of such factors and HS GAG chains present on the EV surface.

In conclusion, here we provide novel evidence that HS GAG chains on the EV surface have an important role in controlling the composition of the bio‐corona of EVs and on the EV‐mediated delivery of multiple growth factors to recipient cells, such as fibroblasts. This study provides novel insight into the importance of HS GAG chains within the EV corona and their role in intercellular communication. The manipulation of vesicular HSPGs and/or their HS GAG chains will likely provide fruitful avenues for future investigation into the complex communication roles of EVs in a variety of tissue contexts.

## 4 Materials and Methods

### 4.1 Cell Culture

PCa cells (DU145; LGC Standards (ATCC), Middlesex, UK) were cultured in RPMI‐1640 media (ThermoFisher Scientific, Gloucester, UK). DU145 cells were maintained in high‐density bioreactor cultures, as described (Mitchell et al. 2008), and used as a source of PCa EVs. Primary fibroblasts (AG0226 [Coriell Institute for Medical Research, New Jersey, USA]) were maintained in DMEM/F12 media (Gibco) and used at passage <10. Fibroblasts were used as EV‐recipient cells in this study and subject to 48 h growth arrest in serum‐free media, prior to further treatment. All media was supplemented with 100 µg/mL streptomycin (Sigma–Aldrich, Dorset, UK), 100 U/mL penicillin (Sigma–Aldrich) and 2 mM L‐glutamine (Sigma–Aldrich). All cell cultures were maintained with 10% FBS depleted of bovine EVs by overnight ultracentrifugation at 100,000 × g (using Quick Seal tubes; 70 Ti fixed angle rotor, κ‐factor of 44, Optima LE‐80K ultracentrifuge; Beckman Coulter, High Wycombe, UK), followed by serial filtration with 0.2 and 0.1 µm vacuum filters (Millipore, Watford, UK).

### 4.2 EV Isolation and HepIII Digestion of EV‐Associated HS GAG Chains

DU145 cell conditioned media was collected from bioreactor flasks weekly. Media was subject to pre‐clearing of cells and debris by centrifugation twice at 400 × *g*, for 6 min at 4°C, prior to further centrifugation at 2000 × *g*, for 15 min at 4°C. Media was then subject to serial filtration with 0.2 and 0.1 µm syringe filters (Millipore) prior to storage at minus 80°C. Pre‐cleared media samples were thawed and EVs isolated by ultracentrifugation on a 30% sucrose/D20 cushion (Clayton et al. 2007). Briefly, media samples were centrifuged on top of a sucrose cushion to isolate EVs at 100,000 × *g* (using Quick Seal tubes; SW32 rotor) for 1.5 h at 4°C. EVs in sucrose were collected and subject to a PBS wash and EVs pelleted at 100,000 × *g* (max) (using Quick Seal tubes; 70 Ti fixed angle rotor) for 1.5 h at 4°C. EVs were resuspended in PBS and stored at –80°C. EV concentration was determined using the MicroBCA protein assay (ThermoFisher Scientific).

For HS GAG chain digestion, EV samples were digested with (0.006 U/mL) heparinase III (HepIII; Amsbio Abingdon, UK) for 3 h at 37°C in heparinase buffer (0.1% Triton, 0.1 M NaCl, 1 mM CaCl_2_, 50 mM 6‐aminohexanoic acid and 50 mM HEPES, at pH 7). EVs were also incubated with heat‐inactivated HepIII as well as heparinase buffer only, to serve as digestion control conditions. After incubation, samples were washed by ultracentrifugation and resuspended in PBS.

### 4.3 Western Blotting and Immuno‐Affinity Assays for Detection of EV Proteins and Confirmation of HS GAG Chain Removal

For EV characterisation, cell lysates were obtained by RIPA buffer and protease inhibitor cocktail (Santa Cruz Biotechnology, Heidelberg, Germany) and protein was quantified by Bradford assay (BioRad, Watford, UK). Equal protein (20 µg) from cell lysates or EVs were loaded per well. Membranes were probed using antibodies for ALIX, TSG101, GRP94 (Santa Cruz) and GAPDH (Novus Biological, Cambridge, UK). HepIII digestion of HS GAG chains was confirmed using 1 µg/mL of anti‐ΔHS antibody (F69‐3G10, Amsbio). All samples were fractionated in 4%–12% NuPAGE Bis‐Tris gradient gels (Invitrogen, ThermoFisher Scientific), electro‐transferred to PDVF membranes (GE Life Sciences, UK). Signals were visualised with enhanced chemiluminescence detection reagent (LI‐COR, Nebraska, USA) and imaged using the C‐DiGit blot scanner (LI‐COR). To access tetraspanin enrichment and examine HepIII digestion of EVs, an immuno‐affinity assay was used, as described previously (Webber et al. 2014). Samples were immobilised onto protein‐binding 96 well plates (Greiner Bio‐One, Stonehouse, UK) and stained using anti‐CD9 (R&D Systems, Biotechne, Abingdon, UK), anti‐CD63 (Bio‐Rad), anti‐CD81 (Bio‐Rad) or anti‐HS (F58‐10E4, [Amsbio]). Detection was achieved using an anti‐IgG‐biotin conjugate (Perkin Elmer) and europium‐conjugated streptavidin (Perkin Elmer, Buckinghamshire, UK). Signal was detected by time‐resolved fluorescence (TRF) on a PHERAstar FS Microplate (BMG Labtech, Aylesbury, UK).

### 4.4 Nanoparticle Tracking Analysis

EV samples were analysed using NanoSight NS300 nanoparticle tracking system (Malvern Instruments). Samples were diluted in particle free water (Fresenius Kabi, Cheshire, UK) to concentrations up to 2 × 10^9^ particles per millilitre, within the linear range of the instrument, and run at a constant flow rate (set to 50) using a NanoSight syringe pump (Malvern Instruments, Malvern, UK). Five videos of 30 s were captured at 25°C using a sCMOS camera system (OrcaFlash 2.8, Hamamatsu Photonics, Japan) and analysed using the NTA 3.1 software (version 3.1 build 3.1.54). Camara sensitivity was set at 14–16 and detection threshold at 1–3. Concentration, size distribution, mean and mode sizes are calculated for the EVs by the software, and concentrations were further used with protein concentrations to calculate the particle:protein (p:p) ratios as a means of quality assurance (Webber and Clayton 2013). Data represents at least three independent experiments.

### 4.5 CRYO‐EM

EVs were adsorbed onto glow‐discharging holey carbon 200‐mesh copper grids (Quantifoil Micro Tools GmbH, Großlöbichau, Germany). Samples were vitrified by rapid emersion into liquid ethane using a Vitrobot (Maastricht Instruments BV, Maastricht, NL). Vitrified samples were imaged at liquid nitrogen temperature using a JEM‐2200FS/CR transmission cryo‐electron microscope (JEOL, Tokyo, Japan) equipped with a field emission gun and operated at an acceleration voltage of 200 kV. The size of the different structures was determined by Image J and data was based on 329 structures counted across 20 different fields of view.

### 4.6 Olink Proximity Extension Assay and Functional Enrichment Analysis

EVs incubated with active HepIII and heat‐inactivated HepIII (control) were lysed in RIPA buffer prior to analysis by proximity extension assay (Olink Bioscience, Uppsala, Sweden). Analysis of EV proteins was achieved Olink's propriety Proseek Panels (*Cardiovascular III*, *Inflammation* and *Oncology II*) each consisting of a 92‐plex array. Data is provided in the form of arbitrary Normalised Protein eXpression (NPX) units on log2 scale, which was then linearised. Data below the limit values of detection were reported by Olink as LOD (limits of detection), and when both samples reported this, analytes were excluded from analysis. Comparison between HepIII digested and control EV samples were made based on fold change and t‐test corrected for multiple testing using Benjamini–Hochberg adjustment. Functional enrichment analysis was performed for identification of biological pathways, potentially regulated by EV‐associated HS GAG chains using FunRich software (version 3.1.4) (Pathan et al. 2015). The output list was generated from pathways with statistically significant *p* value (*p* < 0.05 from corrected p‐values; corrected by Benjamini–Hochberg adjustment).

### 4.7 Fluorescent Labelling of EVs

EV samples were incubated with AlexaFluor‐594, or AlexaFluor‐633, C5 maleimide dye (200 µg/mL; ThermoFisher Scientific), in a total volume of 50 µL PBS, under agitation for 1 h in the dark at room temperature (Roberts‐Dalton et al. 2017). Exosome Spin Columns MW3000 (ThermoFisher Scientific) were rehydrated according to manufacturer's instructions. Unbound maleimide dye was removed from fluorescently labelled EVs by loading Exosome Spin Columns with sample and subsequent centrifugation at 750 × *g* for 3 min at room temperature. A dye control was prepared by loading Exosome Spin Columns with C5 maleimide dye (200 µg/mL, in the absence of EVs, and centrifugation at 750 × *g* for 3 min). EVs were quantified by using a spectrometer (A280; Nanodrop 2000 Spectrometer; ThermoFisher Scientific).

### 4.8 Assessment of EV Uptake

Fibroblasts were cultured until 80% confluent, then serum starved for 24 h, prior to incubation with labelled EVs (25 µg/mL) or dye control for 1 h. For fluorescent microscopy, fibroblasts were washed, fixed with 4% PFA, and permeabilised by incubation with 0.1% Triton x‐100 for 5 min at room temperature. Cells were washed and stained with Actin Green (AlexaFluor 488 phalloidin: Thermo Fisher Scientific) for for 30 min. Nuclei were then labelled using NucBlue Fixed Cell ReadyProbes (DAPI; Thermo Fisher Scientific) as per the manufacturer's instructions. Fibroblasts were visualised using the Axio Observer Z1 microscope platform with a ZEISS Plan Apochromat 63×/1.4 Oil lens. For flow cytometry‐based assessment of EV uptake, EV‐treated fibroblasts were detached by incubation with trypsin. Fibroblasts were washed and resuspended in PBS, prior to quantitation of AlexaFluor 633‐labelled EVs using a FACSverse cytometer (BD Biosciences, Berkshire, UK). Percentage of cells that had taken up EVs (i.e., AlexaFluor 633‐positive) were determined using FACSDIVA software v8.0.1 (BD Biosciences).

### 4.9 Enzyme‐Linked Immunosorbent Assay (ELISA)

Assessment of proteins within EV isolates or fibroblast‐conditioned media were also performed by ELISA (DuoSet ELISA Development System; R&D Systems), with modifications from the manufacture protocol, where the colorimetric readout was substituted by the europium‐streptavidin conjugate, as described previously (Yeung et al. 2018). All plate‐based assays were read on a PHERAstar FS plate reader (BMG Labtech).

### 4.10 Proteome Profiler Arrays

Fibroblasts were seeded in 24 well plates until 80% confluence and then were serum‐starved for 48 h. Cells were then incubated with 200 µg/mL of EVs treated with either active HepIII, heat‐inactivated HepIII or no enzyme, in DMEM/F12 (without FBS). For the RTK array, cells were treated for 2 h before being lysed with RIPA buffer. For the cytokine array, cells were incubated EVs for 72 h, with Golgi‐Stop and Golgi‐Plug (Becton Dickinson, Oxford, UK) added 18 h before lysis, to prevent cytokine secretion. Samples were corrected for protein and both proteome profiler arrays were performed following manufacture instructions, with the exception that the supplied Chemi Reagent Mix was substituted for an enhanced chemiluminescence detection reagent (LI‐COR) prior to imaging using the C‐DiGit blot scanner (LI‐COR).

### 4.11 Statistical Analysis

Statistical analyses were performed using GraphPad Prism 9 software V9.1.0 (Graph Pad, San Diego, CA). Experiments with two experimental groups were evaluated using students *t*‐test. For experiments with more than two experimental groups, statistical analysis was performed using one‐way ANOVA with Tukey post‐hoc test. *p* values of less than 0.05 were considered statistically significant *p < 0.05, **p < 0.01, ***p < 0.001, ****p < 0.0001. Graphs depict mean ± SEM, from one representative experiment of at least three similar experiments, unless stated otherwise.

## Author Contributions

**Sara Veiga**: investigation, data curation, writing – original draft, writing – review & editing. **Alex P. Shephard**: investigation. **Kate Milward**: investigation. **Alex Cocks**: investigation. **Félix Royo**: conceptualization, writing – review & editing, investigation. **Juan M. Falcon‐Perez**: conceptualization, investigation, writing – review & editing, methodology. **Aled Clayton**: conceptualization, methodology, supervision, funding acquisition, writing – original draft, writing – review & editing. **Jason P. Webber**: conceptualization, methodology, data curation, investigation, supervision, funding acquisition, project administration, writing – original draft, writing – review & editing.

## Funding

This work was supported by Prostate Cancer UK through a Movember Foundation *Fellowship* (CDF13‐001) awarded to Jason P. Webber, and a Cancer Research Wales *PhD Studentship* awarded to Jason P. Webber and Aled Clayton (A.C).

## Conflicts of Interest

The authors declare no conflicts of interest.

## Supporting information




**SUPPLEMENTARY FIGURE S1**. Consistency of EV‐associated proteins detected across multiple batches of isolated EVs. **(A)** EVs previously treated with active HepIII, heat‐inactivated HepIII or no enzyme (4ug per condition) were assayed by ELISA to quantify protein detection. The bar graphs show ± SEM of at least 2 independent experiments each based on a different HepIII digestion and represented by circle, square or triangle. Every independent experiment was performed in triplicate wells. (B) Bar graphs show the mean ± SEM of 3 independent experiments (in triplicate wells) each based on a different EV isolation which are represented by circle, square or triangle. The quantification (pg/mL) of protein detected at the surface of DU145 EVs on 8 µg, 4 µg, 2 µg and 1 µg per 100 µL/well is represented.

**SUPPLEMENTARY FIGURE S2**. Tyrosine kinase receptors phosphorylated in fibroblasts treated with control EVs (EVs treated with no enzyme, or heat‐inactivated HepIII) compared with untreated fibroblasts. (A) A phospho‐RTK Proteome Profiler array was performed on cell lysates from fibroblasts incubated for 2 hours with EVs treated with either no enzyme, heat‐inactivated HepIII enzyme, or incubated in media alone (untreated fibroblasts). (B) Densitometry‐based analysis shows receptors with at least 1.5‐fold change in level of phosphorylation following fibroblast incubation with control EVs compared to untreated fibroblasts. (C) Heatmap of the MID (mean integrated density) values detected for receptors with 1.5‐fold change in phosphorylation following fibroblast incubation with control EVs compared to untreated fibroblasts. Receptors that were below detection limits in untreated fibroblasts, and yet were detected following stimulation with EVs, are indicated by the green boxes.

**SUPPLEMENTARY FIGURE S3**. To assess EV uptake, fibroblasts were treated for 1 hour with 25 µg/mL of fluorescently labelled EVs or a control for free dye. Prior to uptake, EVs had been treated with heat‐inactivated HepIII, active HepIII, or no enzyme. **(A)** For visualisation experiments, fibroblasts received either AlexaFluor‐594 labelled EVs (red) or a control for free dye. Cells were fixed with 4% PFA. Actin was labelled with AlexaFluor‐488 phalloidin (green) and nuclei with NucBlueTM (blue). Cells were visualised by fluorescence microscopy and images captured by Axio Observer Z1 microscope with a ZEISS Plan Apochromat 63x/ 1.4 Oil objective. Representative microscopic fields (of 9 per condition) are shown (scale bar = 50 µm). For flow cytometry‐based analysis, fibroblasts received either AlexaFluor‐633 labelled EVs (red) or a control for free dye, for 1 hour. Fibroblasts were detached and resuspended in PBS, prior to detection of AlexaFluor 633‐labelled EVs within fibroblasts using the FACSverse cytometer. Graph shows mean ± SEM percentage of fibroblasts positive for AlexaFluor‐633 signal **(B)** mean ± SEM Median Fluorescence Intensity (MFI) for AlexaFluor‐633 signal **(C)**, from technical triplicates. Similar results were observed across 5 separate experiments. ns = not significant.

**SUPPLEMENTARY FIGURE S4**. Cytokines produced by fibroblasts treated with control EVs (EVs treated with no enzyme, or heat‐inactivated HepIII) compared with untreated fibroblasts. (A) A human cytokine Proteome Profiler array was performed on cell lysates from fibroblasts incubated for 72 hours with EVs treated with either no enzyme, heat‐inactivated HepIII enzyme, or incubated in media alone (untreated fibroblasts). Fibroblasts were incubated with Golgi‐Stop and Golgi‐Plug 18 h before lysis, to prevent cytokine secretion. (B) Densitometry‐based analysis shows cytokines with at least 1.5‐fold change in level of detection following fibroblast incubation with control EVs compared to untreated fibroblasts. (C) Heatmap of the MID (mean integrated density) values detected for cytokines with 1.5‐fold change in detection following fibroblast incubation with control EVs (No enzyme or heat‐inactivated HepIII) compared to untreated fibroblasts. Cytokines that were below detection limits in untreated fibroblasts, and yet were detected following stimulation with EVs, are indicated by the green box.

**Supplementary Table**: jex270146‐sup‐0005‐TableS1.docx

**Supplementary Table**: jex270146‐sup‐0006‐TableS2.docx

## Data Availability

The data that support the findings of this study are available from the corresponding author upon reasonable request.
