# Supplementary figures and images for: Heparan Sulphate Glycosaminoglycan Chains Contribute to the Tethering of Coronal Factors and Are Important for Extracellular Vesicle‐Mediated Fibroblast Activation
Source: J Extracell Biol. 2026 May 9;5(5):e70146. doi: 10.1002/jex2.70146 (PMC13157584; doi:10.1002/jex2.70146)

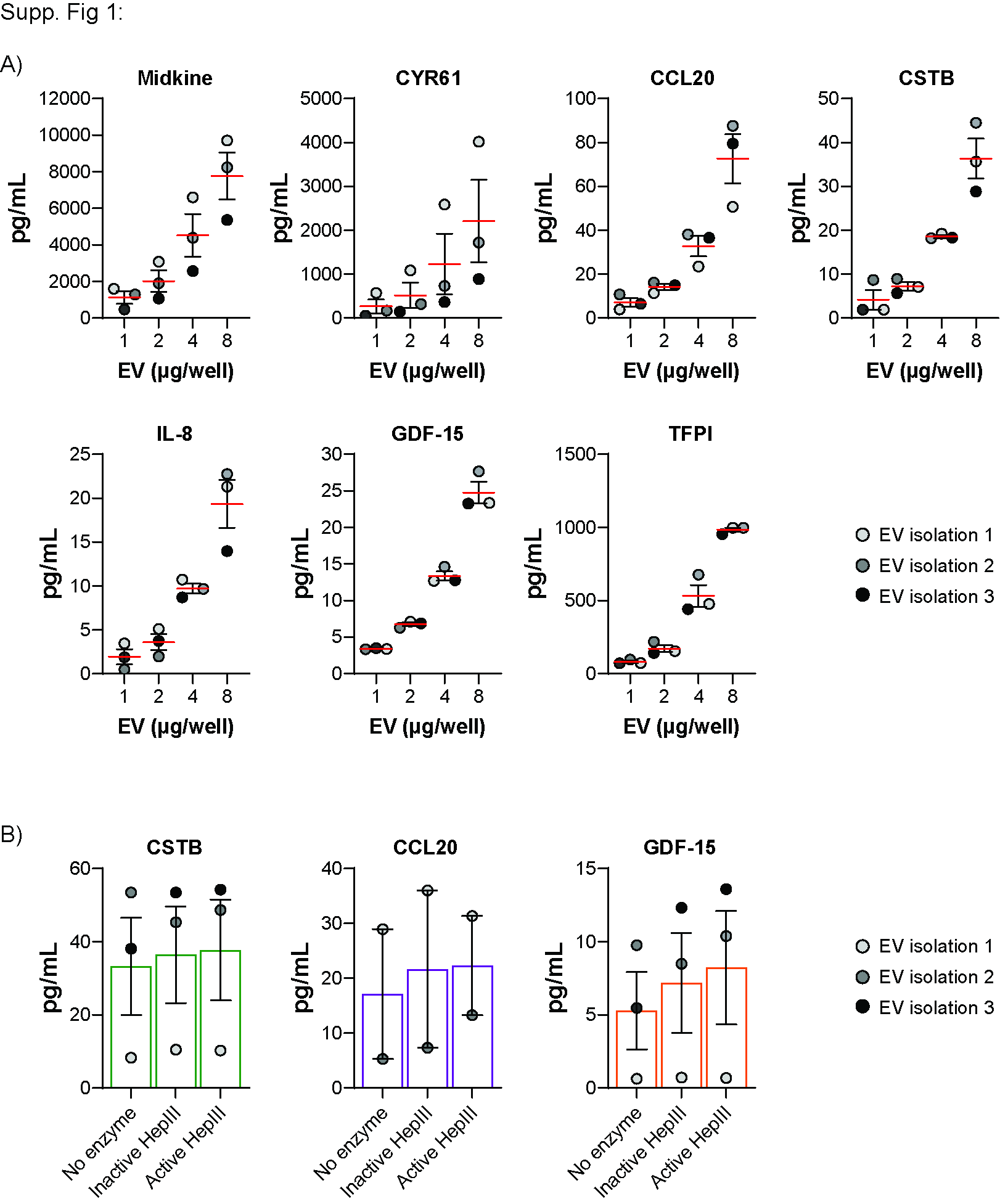

Supplement: Supplementary file 1 — SUPPLEMENTARY FIGURE S1. Consistency of EV‐associated proteins detected across multiple batches of isolated EVs. (A) EVs previously treated with active HepIII, heat‐inactivated HepIII or no enzyme (4ug per condition) were assayed by ELISA to quantify protein detection. The bar graphs show ± SEM of at least 2 independent experiments each based on a different HepIII digestion and represented by circle, square or triangle. Every independent experiment was performed in triplicate wells. (B) Bar graphs show the mean ± SEM of 3 independent experiments (in triplicate wells) each based on a different EV isolation which are represented by circle, square or triangle. The quantification (pg/mL) of protein detected at the surface of DU145 EVs on 8 µg, 4 µg, 2 µg and 1 µg per 100 µL/well is represented. [file JEX2-5-e70146-s006.tif]

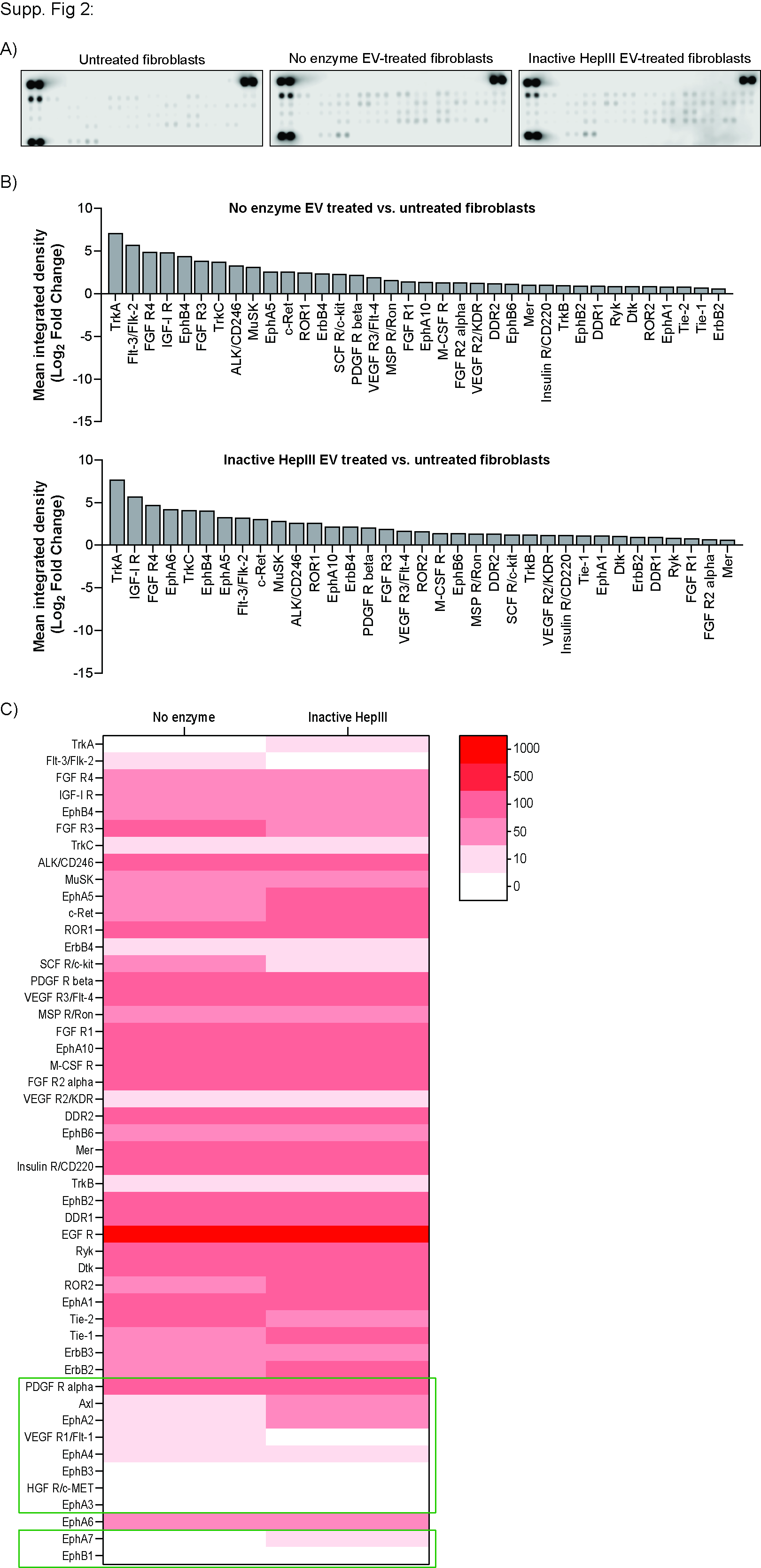

Supplement: Supplementary file 2 — SUPPLEMENTARY FIGURE S2. Tyrosine kinase receptors phosphorylated in fibroblasts treated with control EVs (EVs treated with no enzyme, or heat‐inactivated HepIII) compared with untreated fibroblasts. (A) A phospho‐RTK Proteome Profiler array was performed on cell lysates from fibroblasts incubated for 2 hours with EVs treated with either no enzyme, heat‐inactivated HepIII enzyme, or incubated in media alone (untreated fibroblasts). (B) Densitometry‐based analysis shows receptors with at least 1.5‐fold change in level of phosphorylation following fibroblast incubation with control EVs compared to untreated fibroblasts. (C) Heatmap of the MID (mean integrated density) values detected for receptors with 1.5‐fold change in phosphorylation following fibroblast incubation with control EVs compared to untreated fibroblasts. Receptors that were below detection limits in untreated fibroblasts, and yet were detected following stimulation with EVs, are indicated by the green boxes. [file JEX2-5-e70146-s005.tif]

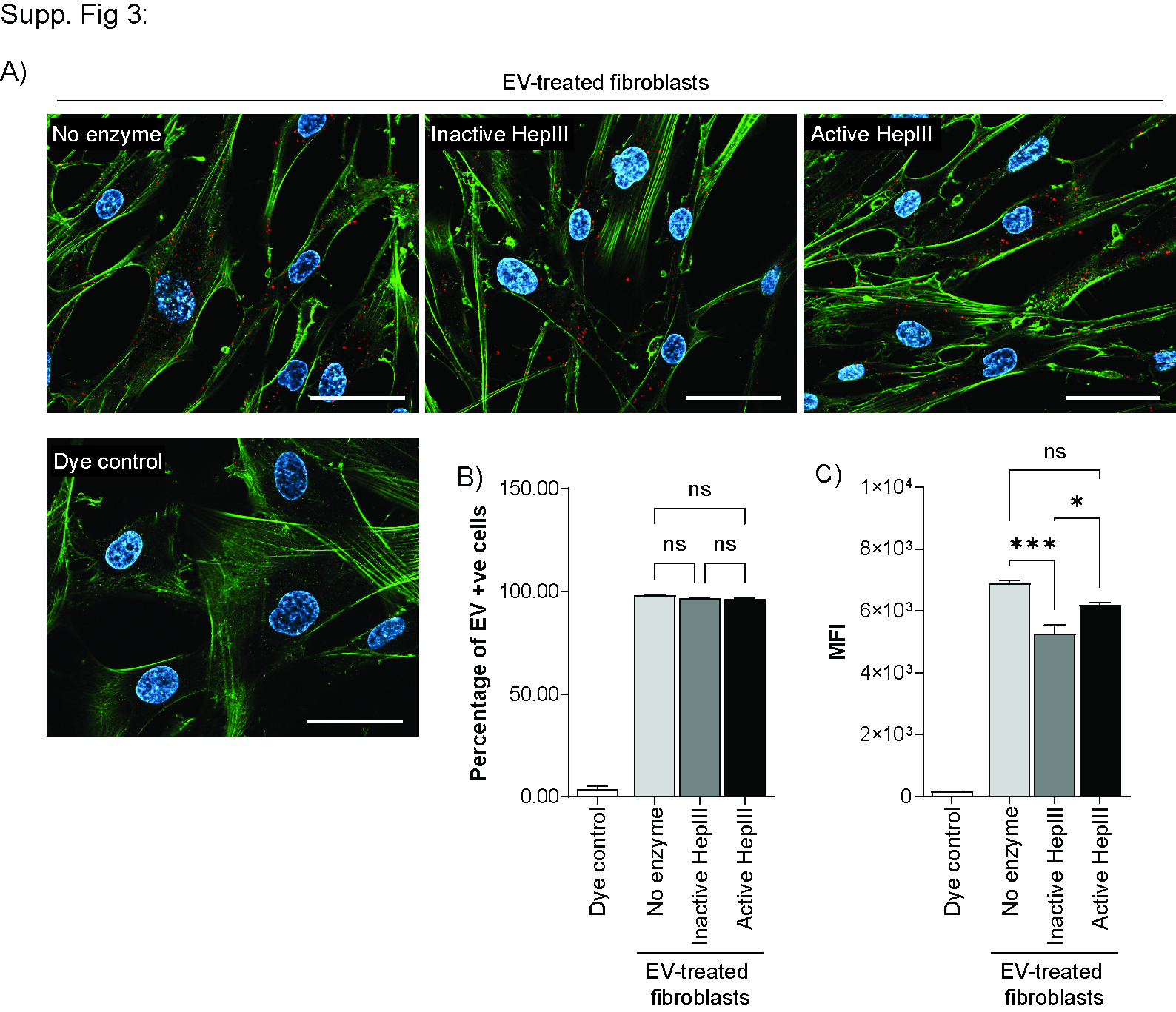

Supplement: Supplementary file 3 — SUPPLEMENTARY FIGURE S3. To assess EV uptake, fibroblasts were treated for 1 hour with 25 µg/mL of fluorescently labelled EVs or a control for free dye. Prior to uptake, EVs had been treated with heat‐inactivated HepIII, active HepIII, or no enzyme. (A) For visualisation experiments, fibroblasts received either AlexaFluor‐594 labelled EVs (red) or a control for free dye. Cells were fixed with 4% PFA. Actin was labelled with AlexaFluor‐488 phalloidin (green) and nuclei with NucBlueTM (blue). Cells were visualised by fluorescence microscopy and images captured by Axio Observer Z1 microscope with a ZEISS Plan Apochromat 63x/ 1.4 Oil objective. Representative microscopic fields (of 9 per condition) are shown (scale bar = 50 µm). For flow cytometry‐based analysis, fibroblasts received either AlexaFluor‐633 labelled EVs (red) or a control for free dye, for 1 hour. Fibroblasts were detached and resuspended in PBS, prior to detection of AlexaFluor 633‐labelled EVs within fibroblasts using the FACSverse cytometer. Graph shows mean ± SEM percentage of fibroblasts positive for AlexaFluor‐633 signal (B) mean ± SEM Median Fluorescence Intensity (MFI) for AlexaFluor‐633 signal (C), from technical triplicates. Similar results were observed across 5 separate experiments. ns = not significant. [file JEX2-5-e70146-s004.tif]

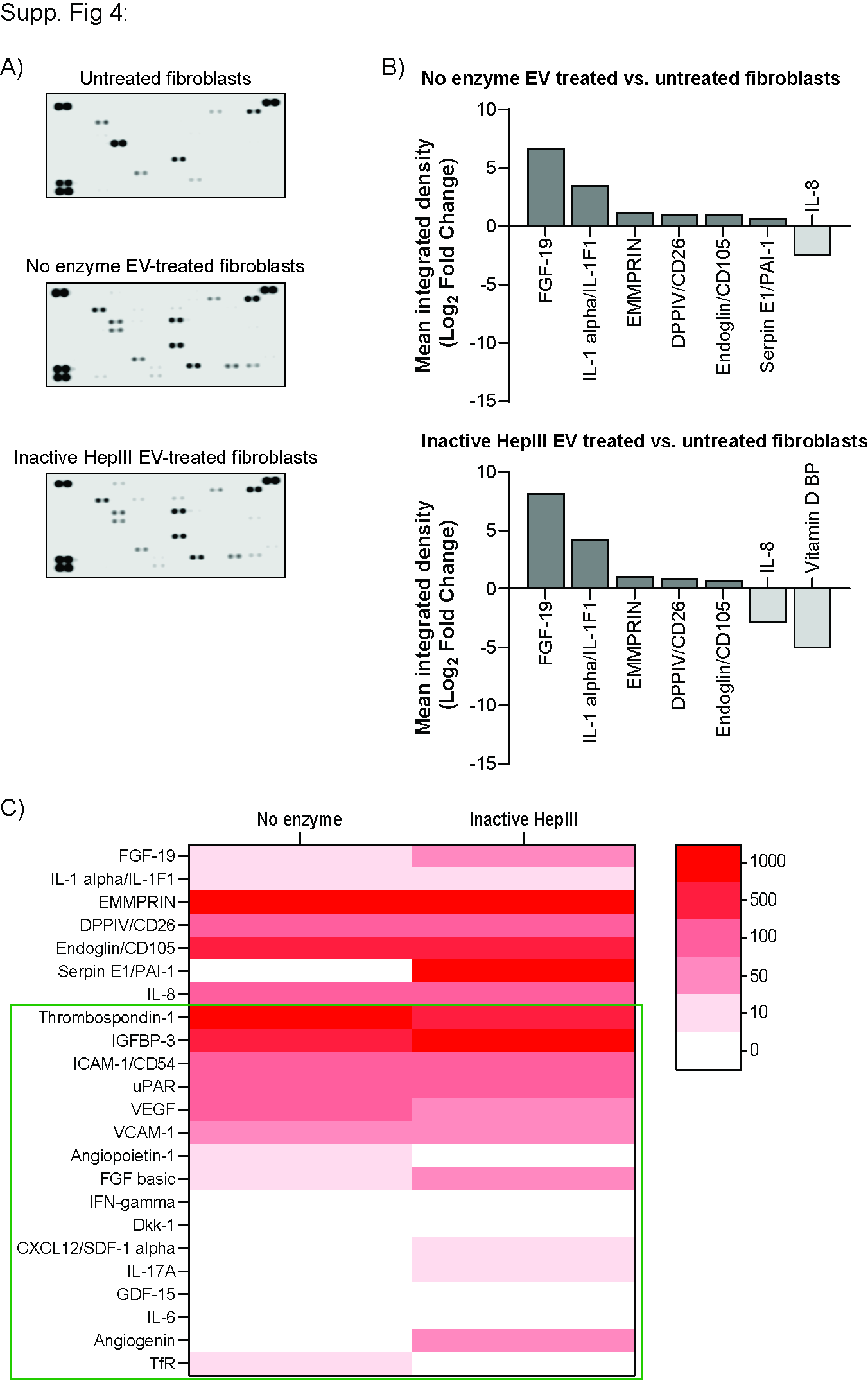

Supplement: Supplementary file 4 — SUPPLEMENTARY FIGURE S4. Cytokines produced by fibroblasts treated with control EVs (EVs treated with no enzyme, or heat‐inactivated HepIII) compared with untreated fibroblasts. (A) A human cytokine Proteome Profiler array was performed on cell lysates from fibroblasts incubated for 72 hours with EVs treated with either no enzyme, heat‐inactivated HepIII enzyme, or incubated in media alone (untreated fibroblasts). Fibroblasts were incubated with Golgi‐Stop and Golgi‐Plug 18 h before lysis, to prevent cytokine secretion. (B) Densitometry‐based analysis shows cytokines with at least 1.5‐fold change in level of detection following fibroblast incubation with control EVs compared to untreated fibroblasts. (C) Heatmap of the MID (mean integrated density) values detected for cytokines with 1.5‐fold change in detection following fibroblast incubation with control EVs (No enzyme or heat‐inactivated HepIII) compared to untreated fibroblasts. Cytokines that were below detection limits in untreated fibroblasts, and yet were detected following stimulation with EVs, are indicated by the green box. [file JEX2-5-e70146-s002.tif]
