# Supplementary material for: Heparan Sulphate Glycosaminoglycan Chains Contribute to the Tethering of Coronal Factors and Are Important for Extracellular Vesicle‐Mediated Fibroblast Activation
Source: J Extracell Biol. 2026 May 9;5(5):e70146. doi: 10.1002/jex2.70146 (PMC13157584; doi:10.1002/jex2.70146)
Supplement: Supplementary file 5 — Supplementary Table: jex270146‐sup‐0005‐TableS1.docx [file JEX2-5-e70146-s003.docx]

**Supplementary Table S1.** List of 48 analytes differentially expressed following HS digestion by HepIII, input into FunRich software for functional enrichment analysis

Cardiovascular III panel Inflammation panel Oncology panel

ALCAM; CSTB; GDF-15; IL-6RA; FAZ; MB; CASP-3; IGFBP-2; GRN

IL-8; MCP-1; CXCL11; AXIN1; CXCL1; CCL4; SCF; IL-18; MMP-1; CXCL5; CXCL6; CXCL10; 4E-BP1; SIRT2; TWEAK; CCL20; ST1A1; STAMPB

TXLNA; CPE; MSLN; TGFR-2; IL-6; TFPI-2; hK8; SCF; CYR61; MetAP 2; PVRL4;

Gal-1; CA9; CTSV; Midkine; ABL1; TLR3; VIM; CXL17; WFDC2; WIF-1
