# Supplementary material for: Heparan Sulphate Glycosaminoglycan Chains Contribute to the Tethering of Coronal Factors and Are Important for Extracellular Vesicle‐Mediated Fibroblast Activation
Source: J Extracell Biol. 2026 May 9;5(5):e70146. doi: 10.1002/jex2.70146 (PMC13157584; doi:10.1002/jex2.70146)
Supplement: Supplementary file 6 — Supplementary Table: jex270146‐sup‐0006‐TableS2.docx [file JEX2-5-e70146-s001.docx]

**Supplementary Table S2.** List of the Top50 most enriched biological pathways identified during Functional Enrichment Analysis

Biological Pathway Genes Mapped (from input data set)

Interleukin-6 signalling IL6R; IL6; PERK regulated gene expression CXCL8; CCL2;

Chemokine receptors bind chemokines

CCL2; CXCL11; CCL4; CXCL10; CCL20;

IL23-mediated signalling events CCL2; CXCL1; IL18; IL6; Signalling by Interleukins IL6R; KITLG; IL18; IL6; Peptide ligand-binding receptors

CXCL8; CCL2; CXCL11; CCL4; CXCL10; CCL20;

AP-1 transcription factor network

Integrin-linked kinase signalling IL6R; CXCL8; CCL2; AXIN1; MMP1; EIF4EBP1; TGFBR2; IL6; CYR61; CA9;

GDF15; IL6R; FAS; CXCL8; CCL2; AXIN1; MMP1;

Glypican pathway

TRAIL signalling pathway

Proteoglycan syndecan-mediated signalling events

Beta1 integrin cell surface interactions

Integrin family cell surface interactions

Insulin Pathway Internalization of ErbB1

Urokinase-type plasminogen activator (uPA) and uPAR- mediated signalling

PDGFR-beta signalling pathway

EGF receptor (ErbB1) signalling pathway

Class I PI3K signalling events Arf6 signalling events

mTOR signalling pathway

Signalling events mediated by focal adhesion kinase

Arf6 downstream pathway S1P1 pathway

ErbB1 downstream signalling Arf6 trafficking events

Class I PI3K signalling events mediated by Akt

EGFR-dependent Endothelin signalling events

IGF1 pathway

GMCSF-mediated signaling events

IL5-mediated signalling events

Signalling events mediated by Hepatocyte Growth Factor Receptor (c-Met)

PDGF receptor signaling network Nectin adhesion pathway

IL3-mediated signalling events

Signalling events mediated by VEGFR1 and VEGFR2

IFN-gamma pathway Glypican 1 network

PAR1-mediated thrombin signalling events

Syndecan-1-mediated signalling events

EIF4EBP1; STAMBP; CPE; TGFBR2; IL6; CYR61; LGALS1; CA9; MDK; ABL1; WIF1;

GDF15; IL6R; FAS; CXCL8; CCL2; AXIN1; MMP1; EIF4EBP1; STAMBP; CPE; TGFBR2; IL6; CYR61; LGALS1; CA9; ABL1; VIM;

GDF15; IL6R; FAS; CXCL8; CCL2; AXIN1; MMP1; EIF4EBP1; STAMBP; CPE; TGFBR2; IL6; CYR61; LGALS1; CA9; MDK; ABL1;

GDF15; IL6R; FAS; CXCL8; CCL2; AXIN1; MMP1; EIF4EBP1; STAMBP; CPE; TGFBR2; IL6; CYR61; LGALS1; CA9; ABL1;

Thrombin/protease-activated receptor (PAR) pathway

Plasma membrane estrogen receptor signalling

VEGF and VEGFR signalling network

Alpha9 beta1 integrin signalling events

Endothelins

LKB1 signalling events

ErbB receptor signalling network

Sphingosine 1-phosphate (S1P) pathway

Alpha6Beta4Integrin

p63 transcription factor network

GDF15; IL6R; FAS; CXCL8; CCL2; AXIN1; MMP1; EIF4EBP1; STAMBP; CPE; TGFBR2; IL6; CYR61; LGALS1; CA9; ABL1;

EIF4EBP1; ABL1; VIM; GDF15; FAS; AXIN1; ABL1;
